# Combinatorial Metabolic Engineering in *Saccharomyces cerevisiae* for the Enhanced Production of the FPP-Derived Sesquiterpene Germacrene

**DOI:** 10.3390/bioengineering7040135

**Published:** 2020-10-24

**Authors:** Jan Niklas Bröker, Boje Müller, Dirk Prüfer, Christian Schulze Gronover

**Affiliations:** 1 Institute of Plant Biology and Biotechnology, University of Muenster, Schlossplatz 8, 48143 Muenster, Germany; j_broe08@uni-muenster.de (J.N.B.); dpruefer@uni-muenster.de (D.P.); 2 Fraunhofer Institute for Molecular Biology and Applied Ecology IME, Schlossplatz 8, 48143 Muenster, Germany; Boje.Mueller@ime.fraunhofer.de

**Keywords:** yeast, metabolic engineering, CRISPR-Cas, farnesyl diphosphate, sesquiterpenes

## Abstract

Farnesyl diphosphate (FPP)-derived isoprenoids represent a diverse group of plant secondary metabolites with great economic potential. To enable their efficient production in the heterologous host *Saccharomyces cerevisiae*, we refined a metabolic engineering strategy using the CRISPR/Cas9 system with the aim of increasing the availability of FPP for downstream reactions. The strategy included the overexpression of mevalonate pathway (MVA) genes, the redirection of metabolic flux towards desired product formation and the knockout of genes responsible for competitive reactions. Following the optimisation of culture conditions, the availability of the improved FPP biosynthesis for downstream reactions was demonstrated by the expression of a germacrene synthase from dandelion. Subsequently, biosynthesis of significant amounts of germacrene-A was observed in the most productive strain compared to the wild type. Thus, the presented strategy is an excellent tool to increase FPP-derived isoprenoid biosynthesis in yeast.

## 1. Introduction

Isoprenoids are found in all living organisms and represent one of the most diverse groups of natural substances. At least 50,000 structures are currently known, many of which have great potential for medical and technical applications [1,2]. Thus far, isoprenoids of low to high molecular weight have been described. While low molecular weight structures such as mono-, sesqui- and triterpenoids are used in the flavour and fragrance industry or as active compounds in pharmaceutical products, high molecular weight compounds such as poly(*cis*-1,4-isoprene), the main component of natural rubber, are widely used for the industrial production of various consumer goods [3,4].

**Figure 1:**
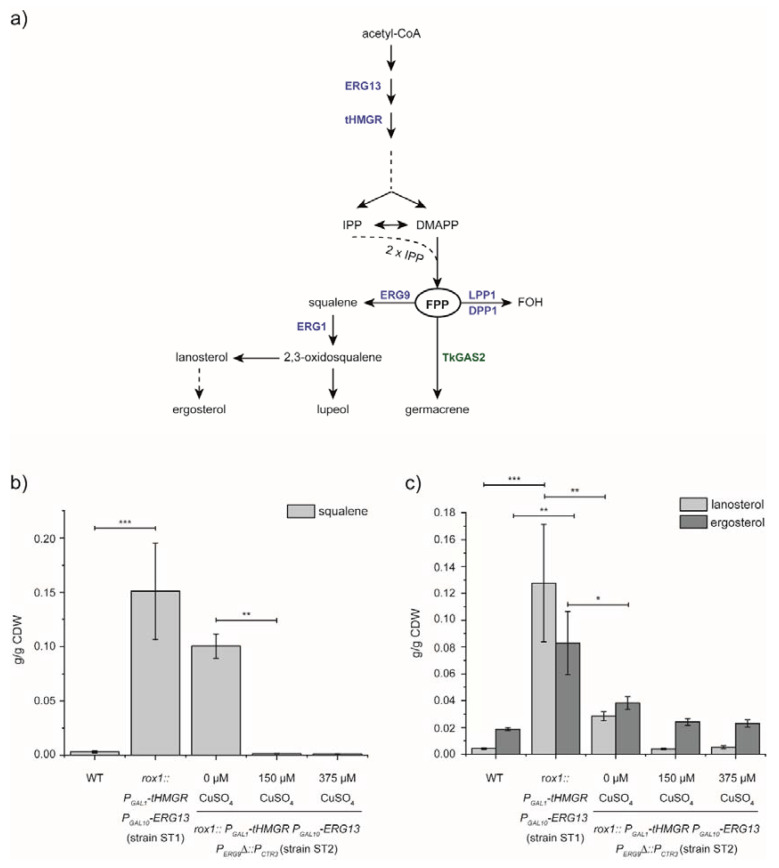
Redirection of MVA-pathway flux. (**a**) Schematic representation of the MVA-pathway leading to FPP-synthesis and downstream metabolites. Dashed arrows represent multiple enzymatic steps. Yeast enzymes are indicated in blue. *T. koksaghyz*-derived enzyme TkGAS2 is indicated in green. HMGS, 3-hydroxy-3-methylglutaryl-coenzyme A (HMG-CoA) synthase; HMGR, 3-hydroxy-3-methylglutaryl-coenzyme A (HMG-CoA) reductase; IPP, isopentenyl diphosphate; DMAPP, dimethylallyl diphosphate; FPP, farnesyl diphosphate; FOH, farnesol; LPP1, lipid phosphate phosphatase 1; DPP1, diacylglycerol pyrophosphate phosphatase 1; TkGAS2, *T. koksaghyz* germacrene-A synthase 2; ERG9, squalene synthase; ERG1, squalene epoxidase. (**b**) Yeast strain ST1 carrying the construct for *tHMGR* and *ERG13* overexpression (*rox1::P_GAL1_-tHMGR P_GAL10_-ERG13*) showed a significantly enhanced squalene accumulation compared to the wild type strain (WT). Yeast strain ST2 carrying the additional copper-sensitive *CTR3* promoter (P_CTR3_) prior to the HMGS coding sequence (strain ST2: *rox1::P_GAL1_-tHMGR P_GAL10_-ERG13 P_ERG9_*∆*::P_CTR3_*) showed a reduced squalene content even without addition of CuSO_4_ to the culture medium. This effect was significantly enhanced upon the addition of 150 and 375 µM CuSO_4_, respectively, indicating the redirection of the metabolic MVA-pathway flux. (**c**) Lanosterol and ergosterol content in the designated yeast strains. Lanosterol and ergosterol content in yeast strains carrying the overexpression cassette for *tHMGR* and *ERG13* (*rox1::P_GAL1_-tHMGR P_GAL10_-ERG13*) showed significantly higher sterol content compared to the WT. Sterol accumulation diminished upon integration of *P_CTR3_* prior to the ERG9 coding sequence. *n* = 3 individual transformants; CDW, cell dry weight; * *p* ≤ 0.05, ** *p* ≤ 0.01, *** *p* ≤ 0.001.

In plants, isoprenoids originate from either the plastidic 2-C-Methyl-D-erythritol 4-phosphate- (MEP) or the highly regulated cytosolic mevalonate (MVA) pathway (Figure 1). In the latter, acetyl-CoA is converted into the isoprenoid building block isopentenyl diphosphate (IPP) and its isomer dimethylallyl diphosphate (DMAPP). The rate limiting step in this pathway is catalysed by the 3-hydroxy-3-methylglutaryl-coenzyme A (HMG-CoA) reductase (HMGR). Once IPP and DMAPP are formed, the sequential condensation of various molecules of IPP to DMAPP by prenyl-transferases (PTs) results in a variety of starter molecules for the synthesis of different isoprenoid backbones. One of these isoprenoid backbones is farnesyl diphosphate (FPP), synthesised from two IPP molecules fused to DMAPP. In the cytosol, this enzymatic reaction is catalysed by farnesyl diphosphate synthase (FPS), which belongs to the enzyme family of *trans*-PTs. FPP then serves as a precursor for the synthesis of, e.g., sesquiterpenes, such as germacrene-A. In addition, dolichols are synthesised by specialised long-chain *cis*-prenyl-transferases (CPTs), resulting in polyisoprenes as well as polyprenols and dolichols of up to C120 [5]. FPP is also discussed as a starter molecule of poly(*cis*-1,4-isoprene) biosynthesis in various rubber producing plant species [6,7,8,9,10].

Due to the growing interest in and demand for isoprene-derived molecules, heterologous hosts such as the yeast *Saccharomyces cerevisiae* are increasingly emerging as alternative production platforms and great efforts have been made to optimise the MVA-pathway in yeast. In this context, strategies such as the overexpression of either *ERG13*, which codes for yeast HMG-CoA synthase (HMGS), or a shortened, deregulated *HMGR* (*tHMGR*) and the deletion of negative regulators such as *ROX1* have been reported [11,12,13,14]. Further increased product titres were obtained by suppression or deletion of genes from competing metabolic pathways. One example is the suppression of essential sterol biosynthesis by replacing of lanosterol synthase promoter (*P_ERG7_*) or squalene synthase promoter (*P_ERG9_*) with the copper-repressible promoter of copper transporter 3 (*P_CTR3_*) [14,15]. Since the repression of *ERG9* was reported to result in a rapid dephosphorylation of the enhanced intracellular FPP pool towards farnesol (FOH), the deletion of phosphatases, which are not essential for regular growth, was used to compete this phenomenon. Therefore, the knockout of phosphatases showing FPP dephosphorylating activity, such as lipid phosphate phosphatase 1 (LPP1) or diacylglycerol pyrophosphate phosphatase 1 (DPP1), was discussed to restore the availability of intracellular FPP for further enzymatic reactions [16,17,18,19,20]. Another effect of FPP hydrolysis to FOH has been described for a squalene synthase (SQS) deficient yeast strain, showing enhanced farnesol formation in response to the initial pH in the cultivation media [21].

In this study, we aimed at the increased accumulation of the intracellular FPP pool and its availability for downstream reactions in yeast. To implement our metabolic engineering strategy, which includes the overexpression of MVA-pathway genes (*tHMGR*; *ERG13*) in combination with the knockout of a negative regulator (*ROX1*), the repression of the competitive sterol biosynthesis by insertion of the copper repressible *CTR3*-promoter prior to the ERG9 coding sequence and the knockout of phosphatases (*LPP1*; *DPP1*), we chose the CRISPR/Cas9 system to avoid the extensive use of auxotrophic markers. In addition, culture conditions were optimised and the availability of the enhanced FPP pool in yeast was demonstrated by the expression of a newly identified *Taraxacum koksaghyz*-derived germacrene-A synthase (*TkGAS2*).

## 2. Materials and Methods

### 2.1. Cloning of Constructs

The construct pESC-*rox1*_*tHMGR*/*ERG13* was generated by digestion of pESC-*rox1*-*KlUra3*_*tHMGR/ERG13* [14] with *Sbf*I and *Bsi*WI followed by ligation after DNA Polymerase I, Large (Klenow) Fragment (New England Biolabs GmbH, Frankfurt a.M., Germany) treatment.

The construct pESC_*P_ERG9__P_CTR3__ERG9* was generated in a two-step process. In a first step, an *ERG9* fragment and a *CTR3* promoter fragment, containing overlapping regions, were amplified using *S. cerevisiae* genomic DNA as a template. For the *ERG9* fragment, forward primer 5′-*CACATTTAAGGGCTATACAAAG*ATGGGAAAGCTATTACAATTG-3′ and reverse primer 5′-AAAGCGGCCGCGTCGTGGACGGTTTGCAA-3′ were used. For the *CTR3* promoter fragment, 5′-AAAGCGGCCGCCAGCTGAAGGATCCGGTATTCCAATGAGAATCGC-3′ and reverse primer 5′-*CCAATTGTAATAGCTTTCCCAT*CTTTGTATAGCCCTTAAATGT-3′ (italic letters indicate the overlapping region, while restriction sites are underlined) were used. The products were fused by overlapping PCR using the *CTR3* promoter forward primer and the *ERG9* reverse primer. The spliced product was digested with *NotI* and transferred to the pESC-*URA* vector linearised with the same enzyme to obtain pESC_*P_CTR3__ERG9*. In the second step, the upstream *ERG9* promoter fragment was amplified from *S. cerevisiae* genomic DNA using forward primer 5′-AAACAGCTGTTGCCTATGCTTTGTTTGC-3′ and reverse primer 5′-AAAGGATCCCTCCCAGAACCCACCGG-3′, and it was transferred to the *Pvu*II/*Bam*HI sites of pESC_*P_CTR3__ERG9* to obtain pESC_*P_ERG9__P_CTR3__ERG9*.

To generate guide RNA and *Cas9* expressing plasmids pML104_*ROX1*, pML104_*ERG9*, pML104_*LPP1* and pML104_*DPP1,* the Cas9 coding sequence containing plasmid pML104 was used [22]. Therefore, the 20mer guide sequences were generated using the ATUM CRISPR gRNA Design tool and the corresponding oligonucleotides to generate the guide RNA expression cassette (Table S1) were designed as described previously [22]. Hybridisation of the oligonucleotides was performed by heating of the reaction mixture to 95 °C for 10 min. Then, the samples were cooled at room temperature for at least 30 min. Hybridised oligonucleotides were ligated into pML104 using T4 ligase to obtain the corresponding plasmids. The same procedure was used to obtain the hybridised oligonucleotides used for the integration of premature stop codons and modified PAM, to prevent further Cas9 activity (Table S1). To check the integration of the stop codon, the corresponding genomic region was sequenced after amplification using proof reading polymerase containing Phusion Green Hot Start II High-Fidelity PCR Master Mix (ThermoFisher Scientific, Waltham, MA, USA).

The construct pAG305_*P_GAL1_-TkGAS2* was prepared by amplifying the TkGAS2 coding sequence from *T. koksaghyz* cDNA using forward primer 5′-AAAGGATCCATGGCTCTAGTTAGAAAC-3′ and reverse primer 5′-AAAGCGGCCGCTTAGCAGCTTTCAAG-3′ (restriction sites are underlined). The PCR product was purified, digested and inserted into plasmid pENTR4 (Invitrogen, Carlsbad, CA, USA). The TkGAS2 coding sequence was then introduced into pAG305-*P_GAL1_-ccdB* (Addgene, Cambridge, MA, USA) [23] by LR recombination to obtain pAG305_*P_GAL1_-TkGAS2*.

The integrity of all constructs was verified by sequencing [24] on an ABI PRISM 3100 Genetic Analyzer (Applied Biosystems, Foster City, CA, USA). Restriction enzymes were obtained from New England Biolabs GmbH.

### 2.2. Strain Construction and Culture Conditions

The *S. cerevisiae* strain CEN.PK2-1C (obtained from EUROSCARF, Oberursel, Germany) was transformed using the lithium acetate method [25] with *URA3* (pML104 constructs) and *LEU2* (pAG305_PGAL1-TkGAS2) as selectable markers. For stable integration into the yeast genome, pESC-*rox1_tHMGR/ERG13* and pESC_*P_ERG9__P_CTR3__ERG9* were digested with *Not*I to remove the plasmid backbone and the corresponding fragments were co-transformed with pML104_*ROX1* and pML104_*ERG9*, respectively. For stop codon integration, 10 µL of the corresponding hybridised oligonucleotides containing the additional stop codon were co-transformed with pML104_*LPP1* and pML104_*DPP1*, respectively.

For the genomic integration into *LEU2*, pAG305_*P_GAL1_-TkGAS2* was linearised with *Bst*EI prior to transformation.

Following transformation, the yeast cells were plated on minimal synthetic defined (SD) medium lacking the corresponding amino acids and/or nucleotide supplements (Clontech, Mountain View, CA, USA) and incubated at 30 °C. Clones were checked for integrity by colony PCR using primers spanning both ends of the integrated constructs if needed. pML104 plasmids were removed via counter-selection on 5-fluoroorotic acid (5-FOA).

For the expression of galactose-inducible genes, a single colony was picked, inoculated into 5 mL yeast extract peptone dextrose (YPD) medium and cultivated overnight at 30 °C on a rolling platform. From this culture, 50 mL of fresh YPD medium (containing 150 µM CuSO_4_ when repressing the expression of *ERG9*) were inoculated to a an OD_600nm_ of 0.2 and incubated at 30 °C shaking at 140 rpm in a 250-mL Erlenmeyer flask. When the culture reached OD_600nm_ of 0.4, the medium was changed to yeast extract peptone galactose medium to induce gene expression. To check the functionality of the *tHMGR* and *ERG13* overexpression as well as *ERG9* repression cells were grown for 19 h and harvested by centrifugation (10 min, 1000× *g*). For germacrene analysis 10% [*v*/*v*] n-dodecane was added to the cultures growing in galactose containing media for 48 h to capture the germacrene and other FPP derivates in the organic layer.

### 2.3. GC-MS Analysis

For the measurements of yeast sterols, metabolites were extracted as described by Rodriguez et al. (2014) [26]. Briefly, freeze-dried yeast cells were incubated at 80 °C in a water bath for 5 min after adding 1 mL 6% [*w*/*v*] KOH in methanol (Carl Roth, Karlsruhe, Germany) and 100 µg cholesterol as an internal standard (Sigma, St. Louis, MI, USA). To extract the metabolites from the methanol mixture, 1 mL of n-hexane (Carl Roth) was added. After vortexing, the upper phase was transferred to a new vial and the extraction was repeated two times using 500 µL n-hexane. The n-hexane of the pooled extracts was removed by evaporation. Samples were re-solubilised in 1 mL acetone (Carl Roth) prior to gas chromatography mass spectrometry (GC-MS) analysis.

For germacrene measurements, 100 µL of the organic layer were transferred to 900 µL n-hexane prior to GC-MS analysis [27]. GC-MS was conducted on a GC-MS-QP 2010 Ultra (Shimadzu, Duisburg, Germany) equipped with a 30 m Rtx-5MS column. After a 1 min hold at 120 °C, the temperature was increased to 330 °C at 21 °C/min (pressure = 58.8 kPa) followed by a hold at 330 °C for 10 min for the analysis of sterols. Germacrene measurements were performed with a 1 min hold, followed by an increasing temperature to 180 °C at 25 °C/min. After a 5 min hold at 180 °C, the temperature was increased to 300 °C at 15 °C/min and was held constant for 3 min. The last temperature increase to 330 °C was performed at 75 °C/min and was held for 5 min (pressure = 36.7 kPa). Different compounds were identified according to their ion mass/charge ratios (41, 55, 69, 93, 95, 105, 109, 121, 161, 189, 204, 207, 218, 271, 285 and 411 m/z) by peak integration using LabSolution software (Shimadzu) and matching to the National Institute of Standards and Technology library. Germacrene was identified as β-elemene, due to the injection temperature of 240 °C [28]. The total ion current of the detected substances was normalised against the internal standard cholesterol and the dry weight of the corresponding sample. The statistical significance of the results was confirmed using ANOVA with the post-hoc Tukey’s honest significant difference test.

## 3. Results

### 3.1. Manipulation of the MVA-Pathway by Overexpression, Deletion and Repression of Pathway Genes and Regulators

In a previous study, we produced the plant triterpene lupeol in yeast. The last steps of lupeol synthesis starting from FPP are the formation of squalene by SQS, which is further converted by the enzyme squalene epoxidase into 2,3-oxidosqualene and then cyclised by lupeol synthases. To improve lupeol biosynthesis in *S. cerevisiae*, we overexpressed the *ERG13* and *tHMGR* genes of the MVA-pathway. In parallel, we inhibited the expression of the negative regulator *ROX1* of the MVA-pathway and sterol biosynthesis [14]. For this purpose, an expression cassette consisting of the coding sequences for ERG13 or tHMGR controlled by the bidirectional *GAL1/GAL10* promoter (*P_GAL1_; P_GAL10_*) was flanked by homologous sequences to the *ROX1* target sequence to increase the integration efficiency by CRISPR/Cas9. Overexpression of *tHMGR* and *HMGS* in the genetic background of *ROX1*-knockout resulted in an increased accumulation of 0.1509 g/g CDW squalene, 0.1276 g/g CDW lanosterol and 0.0829 g/g CDW ergosterol (Figure 1b) in the corresponding yeast strain ST1 (rox1::*P_GAL1__tHMGR P_GAL10_-ERG13*; Table S2) compared to 0.032 g/g CDW squalene, 0.042 g/g CDW lanosterol and 0.0187 g/g CDW ergosterol in the wildtype strain CEN.PK2-1C (WT). The minor amounts of 2,3-oxidosqualene were almost unaffected (Table S2). Although we wanted to increase the flux of the MVA-pathway towards an accumulation of FPP, we targeted the suppression of *ERG9* to prevent the increased pathway activity from not being made available for competitive and preferred squalene synthesis and downstream sterol biosynthesis. Therefore, we inserted a previously described promoter fragment from the *CTR3* gene (*P_CTR3_*) upstream of the endogenous ERG9 coding sequence of the yeast strain that accumulates squalene leading to strain ST2 (Table S2) [14,15]. Additionally, the integration resulted in the deletion of a 327-bp fragment of the endogenous *ERG9* promoter. To verify the success of the metabolic engineering, positive yeast colonies were grown in the presence of 0, 150 and 375 µM CuSO_4_ and expression of *tHMGR* and *ERG13* was induced with galactose (Figure 1b; strain ST2). In comparison to strain ST1, smaller amounts of squalene (0.1004 g/g CDW) were detected without copper addition, while a more significant decrease was observed in the presence of 150 µM CuSO_4_ (0.0015 g/g CDW), and it was 0.0013 g/g CDW at 375 µM CuSO_4_ (Figure 1b). In addition, we observed an increase in 2,3-oxidosqualene with suppression of *ERG9*, suggesting upregulation of squalene epoxidase *ERG1* to ensure supply of downstream sterols (Table S2). However, levels of downstream sterols decreased significantly without the addition of copper as representatively shown for lanosterol (0.0283 g/g CDW) and ergosterol (0.0382 g/g CDW). Since the addition of 150 µM CuSO_4_ in the culture media resulted in a significant decrease of squalene content, we chose this concentration for the following experiments.

### 3.2. Expression of TkGAS2 to Validate the Potential of Sesquiterpenoid Production of the Engineered Yeast Strain

**Figure 2:**
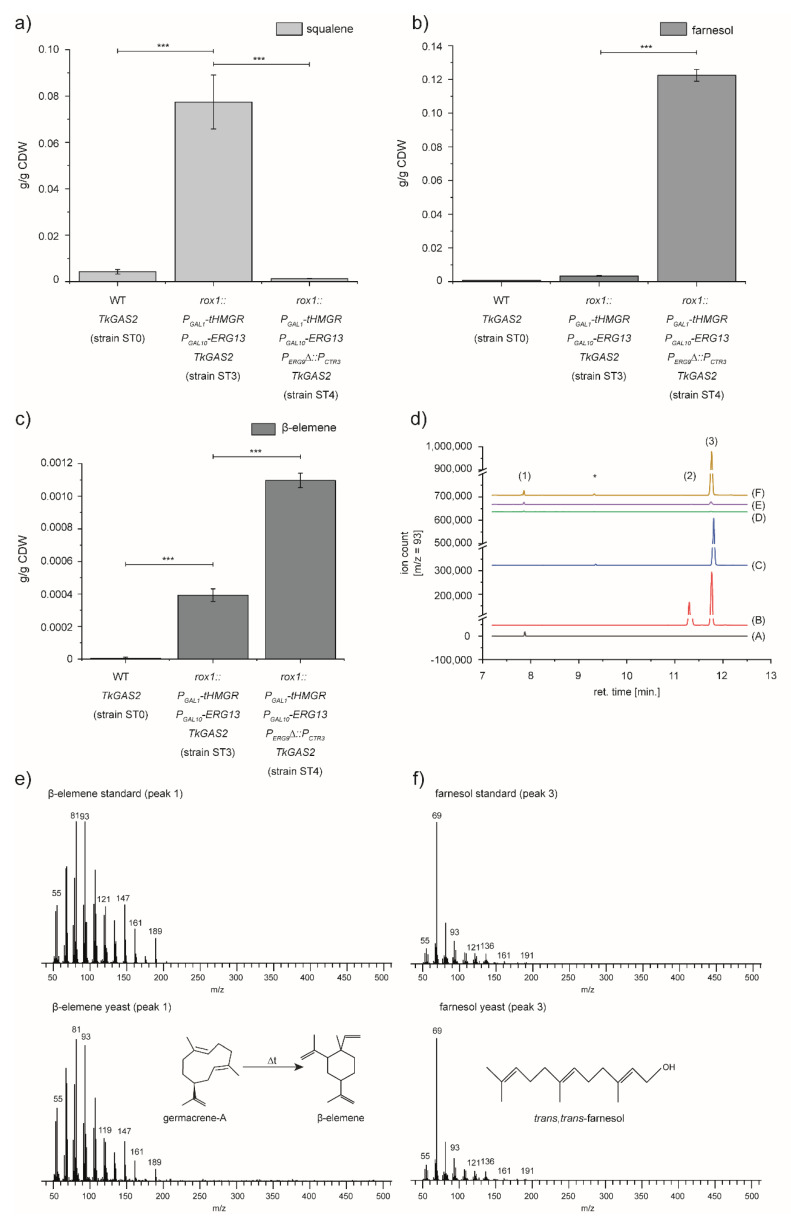
Germacrene-A and farnesol synthesis in *S. cerevisiae* expressing *TkGAS2*. (**a**) Squalene accumulation in yeast strains expressing *TkGAS2*. Yeast strain ST3 carrying the TkGAS2 coding sequence in addition to the *HMGR* and *ERG13* overexpression cassette (*rox1::P_GAL1_-tHMGR P_GAL10_-ERG13 TkGAS2*) showed a significantly increase in squalene accumulation compared to the wild type yeast expressing *TkGAS2* (strain ST0). This accumulation diminishes upon introduction of the *CTR3* promoter prior to the ERG9 coding sequence (strain 4) and in the growth of CuSO_4_ containing media. (**b**) Enhanced *trans-trans-*farnesol accumulation upon *ERG9* repression. *trans-trans-*farnesol starts to accumulate significantly upon repression of *ERG9* (strain 4). (**c**) Germacrene-A is converted into β-elemene, caused by the high temperatures during GC-MS analysis [27,28] and accumulates continuously in the engineered yeast strains ST3 and ST4. (**d**) Yeast strains carrying the TkGAS2 coding sequence ((D) = WT *TkGAS2* (strain ST0); (E) = *rox1::P_GAL1_-tHMGR P_GAL10_-ERG13 TkGAS2* (strain ST3); (F) = *rox1::P_GAL1_-tHMGR P_GAL10_-ERG13 P_ERG9_*∆*::P_CTR3_ TkGAS2* (strain ST4)) showed an additional peak in the GC-MS spectrum (m/z = 93; peak (1)) compared to the control strain ((C) = *rox1*::*P_GAL1_-tHMGR P_GAL10_-ERG13 P_ERG9_∆::P_CTR3_* (strain ST2)). The peak most likely represents β-elemene (ret. time = 7.9 min) as it matched to the corresponding standard ((A)). The significantly enhanced peak that most likely represents *trans-trans-*farnesol (peak (3); ret. time = 11.855 min) could be identified in the n-dodecane layer of yeast cultures upon ERG9 repression, as well as in the corresponding standard (B). peak (1) = β-elemene; peak (2) = *cis*-*trans*-farnesol and *cis*-*cis*-farnesol; peak (3) = *trans*-*trans*-farnesol; asterisk = unidentified yeast metabolite. (**e**) Mass spectra of the β-elemene standard and the designated β-elemene from yeast cultures, formed upon thermal conversion of germacrene-A in the analytic process. (**f**) Mass spectra of the *trans*-*trans*-farnesol standard peak and the designated *trans-trans-*farnesol peak produced by yeast cultures. *n* = 3 individual transformants; CDW, cell dry weight; *** *p* ≤ 0.001.

The elevated FPP levels in yeast can be utilised to produce a broad range of valuable sesquiterpenoids. As an example, we chose germacrene-A, a well-described sesquiterpene suitable for GC-MS measurements, because measurement of MVA intermediates remains challenging [29]. For the identification of a germacrene-A synthase in our model plant *T. koksaghyz*, we screened a RNA-seq database with a germacrene-A synthase sequence from the closely related dandelion species *T. officinale* (*ToGAS2*) [30]. In subsequent experiments, we were able to obtain a 1755-bp open reading frame, encoding a polypeptide of 584 amino acids that showed 99.3% sequence identity to *ToGAS2* [30]. The resulting gene was designated as *TkGAS2*. For the expression of *TkGAS2* in yeast, we inserted the *TkGAS2* gene into pAG305_*P_GAL1_* to enable its recombination into the *LEU2* locus of the yeast genome. Compared to yeast strains ST1 and ST2 (Figure 1), GC-MS analysis identified a similar squalene pattern for the recombinant TkGAS2 yeast strains ST3 (cf. ST1) and ST4 (cf. ST2) after cultivation (Figure 2). Furthermore, we measured the diluted n-dodecane layer that was used to trap the potential product of *TkGAS2* and could observe an additional peak in the GC-MS spectra of the *TkGAS2* expressing yeast cultures ST0, ST3 and ST4, but not in control strain ST1 or ST2. The peak, at a retention time of 7.9 min, was identified as β-elemene by the comparison to the measurement of an authentic β-elemene standard. As a described thermal conversion of germacrene-A to β-elemene occurs during the GC-MS measurements [28,31], the identification of β-elemene supported our annotation of the *TkGAS2* sequence. Another additional peak was observed in the extract of the strains harbouring the constructs for *HMGS* and *tHMGR* (over-)expression in combination with the construct for the repression of sterol biosynthesis (strain ST2 and ST4). The peak with a retention time of 11.855 min was also compared to the measurement of an authentic standard and therefore identified as the alcoholic FPP derivate *trans*-*trans*-farnesol. This peak reflects the previously described hydrolysis of FPP to farnesol upon ERG9 repression, caused by phosphatases such as LPP1 and DPP1 or by non-enzymatic hydrolysis [16,21].

Based on these results and increasing germacrene-A synthesis up to 0.0011 g/g CDW (strain ST4), we concluded that the two FPP products *trans-trans-*farnesol and germacrene-A are the result of improved FPP synthesis caused by the redirection of MVA-pathway flow.

### 3.3. Enhanced Germacrene-A Synthesis by Preventing FPP Hydrolysis

**Figure 3:**
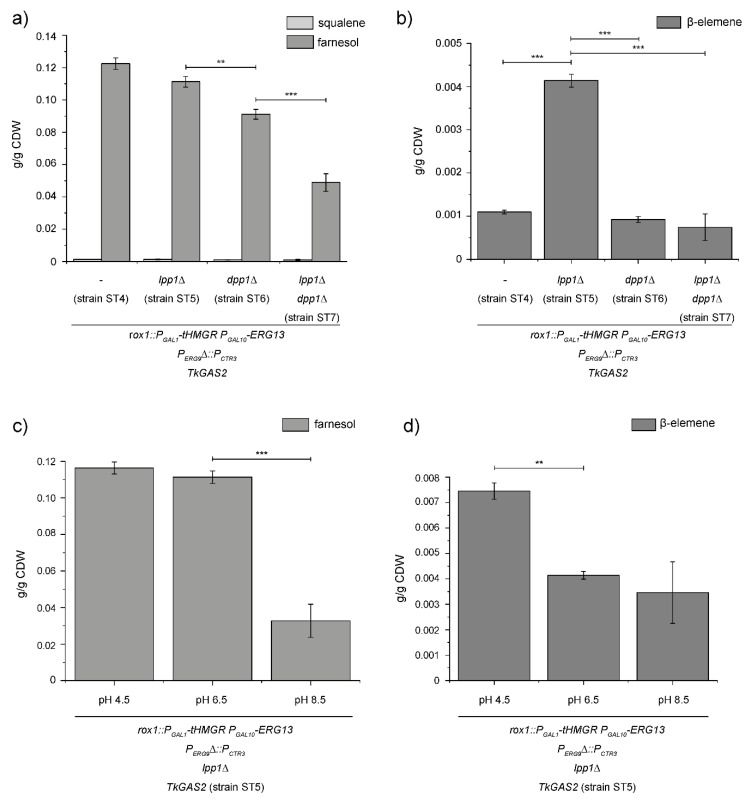
Enhanced germacrene-A synthesis by inhibition of FPP degradation. (**a**) Squalene and *trans-trans-*farnesol levels in yeast strains harbouring the phosphatase knockouts. Yeast strains carrying the premature stop codon in the LPP1 (*lpp1*∆, strain ST5) and/or DPP1 (*dpp1*∆, strain ST6; *lpp1*∆ *dpp1*∆, strain ST7) coding sequence showed comparable squalene levels as the control strain ST4, confirming the repression of sterol biosynthesis pathway upon addition of 150 µM CuSO_4_. The yeast strain carrying the single *LPP1* knockout showed a slightly reduced synthesis of *trans-trans-*farnesol (strain ST5). A more significant decrease was detected for the strain carrying the *DPP1* knockout (strain ST6). An additional effect of the double knockout could be observed in the corresponding mutant (strain ST7). (**b**) Germacrene-A synthesis of yeast strains harbouring the phosphatase knockouts. In contrast to the continuously decreasing *trans-trans-*farnesol content in the phosphatase knockout yeast strains, an enhanced synthesis of germacrene-A could only be detected in the *LPP1* single knockout strain (strain ST5). (**c**) t*rans-trans-*farnesol content after cultivation of yeast under different pH conditions. To test the influence of the initial media pH towards the hydrolysis of FPP to *trans-trans-*farnesol, the yeast strain optimised for germacrene-A synthesis was cultured in media with an initial pH of 4.5, 6.5 and 8.5, whereas almost no effect could be detected under acidic pH conditions (pH 4.5). A significantly decrease in *trans-trans-*farnesol production was observed after culturing the strain in alkaline media (pH 8.5). (**d**) β-elemene content after cultivation under different pH conditions. An almost doubled β-elemene synthesis could be observed in the strain cultured under acidic conditions (pH 4.5), whereas no significant change in β-elemene synthesis could be detected upon cultivation in alkaline media (pH 8.5). *n* = 3 individual transformants; CDW, cell dry weight; ** *p* ≤ 0.01, *** *p* ≤ 0.001.

Next, we aimed to improve the intracellular FPP pool by inhibition of FPP dephosphorylation to further optimise the yeast strain ST4. Therefore, we chose *LPP1* and *DPP1* as targets to prevent the enzymatic degradation of FPP, as they were described to be responsible for most of the dephosphorylation activity towards phosphorylated isoprenoids and were used in previous studies that aimed for enhanced sesquiterpene synthesis, but with equivocal success [16,17,18,19,20]. Consequently, we used the CRISPR/Cas9 system to integrate premature stop codons into the corresponding loci to prevent translation of the phosphatase encoding genes and to set up the corresponding single and double mutant strains ST5, ST6 and ST7 (*rox1::P_GAL1_-tHMGR P_GAL10_-ERG13 P_ERG9_*∆*::P_CTR3_ TkGAS2 lpp1*∆; *dpp1*∆; *lpp1*∆ *dpp1*∆; Table S2; for the corresponding sequence data, refer to Figure S1). After cultivation and GC-MS analysis, only minor amounts of squalene could be detected, ranging from 0.001 g/g CDW in the double knockout strain ST7 (Figure 3a) to 0.0013 g/g CDW in the strain ST5 carrying the LPP1 knockout, which was in accordance with our previous measurements upon overexpression of *tHMGR* and *ERG13* in combination with the repression of sterol biosynthesis (0.0013 g/g CDW; strain ST4; Figure 2a,b). Furthermore, we could detect decreased amounts of *trans*-*trans*-farnesol (peak 3 in Figure 2d) in the yeast strains carrying the phosphatase knockouts with the smallest decrease from 0.1225 g/g CDW in the preceding strain ST4 towards 0.1113 g/g CDW in the *LPP1* knockout strain ST5. A more severe effect was observed upon the knockout of *DPP1* (0.0911 g/g CDW; strain ST6) as well as in the double mutant (strain ST7) that produced 0.0489 g/g CDW of *trans-trans*-farnesol. However, these results only confirmed our expectation of a proportional increase in germacrene-A synthesis with respect to the yeast strain ST5 carrying the *LPP1* knockout, which produced 0.0041 g/g CDW of β-elemene (peak 1 in Figure 2d) compared to 0.0011 g/g CDW obtained for the strain ST4 lacking any phosphatase knockout (Figure 3b). The amount of β-elemene is proportional to germacrene-A in the dodecane layer, because by temperature-dependent rearrangement during GC-MS analysis [27,28].

As we could decrease the enzymatic degradation of FPP to *trans-trans-*farnesol by the knockout of *LPP1*, we further aimed to decrease hydrolysis of FPP by optimisation of the culture media, allowing its intracellular accumulation for downstream reactions as e.g., germacrene-A synthesis. As Muramatsu and colleagues reported in 2008 [21], FPP hydrolysis was the basis for increased farnesol production in an *ERG9*-deficient yeast strain and is dependent on the pH value of the culture medium. Therefore, we tested different initial pH values of the culture media ranging from pH 4.5 to pH 8.5 and measured the *trans-trans-*farnesol and β-elemene content in the n-dodecane layer (Figure 3c,d). Thereby, we could detect a decreased *trans-trans-*farnesol content of 0.0328 g/g CDW produced by the yeast cultured at an initial pH of 8.5 in contrast to 0.1113 g/g CDW *trans-trans-*farnesol produced by the strain cultured at an initial pH of 6.5, whereas the germacrene-A synthesis was not significantly affected (0.0035 g/g CDW). The *trans-trans-*farnesol production of the yeast cultured under an acidic initial pH of 4.5 did not show a significant change in *trans-trans-*farnesol production (0.1163 g/g CDW), which is in accordance to the most comparable measurements of Muramatsu and colleagues [21]. However, the acidic pH almost doubled the germacrene-A synthesis of our engineered strain (0.0075 g/g CDW) compared to the strain cultured under the previously used conditions (pH 6.5; 0.0041 g/g CDW), which may reflect the decrease of FPP hydrolysis.

## 4. Discussion

In this study, we provided a new approach for improved biosynthesis of FPP-derived isoprenoids by increasing the availability of the intracellular FPP pool in the heterologous host *S. cerevisiae*. Therefore, we successfully expanded our previously described strategy [14] by the use of the CRISPR/Cas9 system to increase and redirect the flux through the MVA-pathway and sterol biosynthesis, respectively.

As expected, the overexpression of *tHMGR* and *ERG13*, as well as the deletion of the negative MVA-pathway regulator *ROX1*, resulted in the enhanced accumulation of squalene and downstream sterols (Figure 1). Then, we integrated the copper-repressible *CTR3*-promoter prior to the ERG9 coding sequence, to enable the redirection of the enhanced MVA-pathway flux. This strategy was previously described to enhance isoprenoid biosynthesis in yeast, including the synthesis of the FPP-derived artemisinin precursor artemisinic acid [15]. Consequently, the addition of CuSO_4_ to the cultivation media of the corresponding strain led to decreased squalene levels (Figure 1), already pointing towards a redirection of the enhanced MVA-pathway flux and an enhanced availability of FPP for downstream reactions.

As several germacrene-A synthases have been previously described and partially been expressed in yeast [27,30], we mined *T. koksaghyz* RNA-seq data for a germacrene-A synthase, representing an enzyme capable of synthesising a product in a stoichiometric equal manner to its FPP consumption and thus, can confirm the availability of the potentially enhanced FPP pool. Upon expression of *TkGAS2* in the corresponding yeast strains, GC-MS measurements revealed the enhanced synthesis of the single enzyme product germacrene-A (Figure 2). Thus, we could confirm our assumption of the availability of the enhanced FPP pool and furthermore represent, to the best of our knowledge, the first functional characterisation of a *T. koksaghyz* sesquiterpene synthase, namely TkGAS2.

Besides the enhanced synthesis of germacrene-A, we observed the accumulation of the alcoholic FPP derivate farnesol in the yeast strain expressing *TkGAS2* upon *ERG9* repression, a phenomenon previously described (Figure 2) [32,33]. Thus, we aimed to prevent the hydrolysis of FPP to *trans-trans-*farnesol to further increase FPP availability for downstream reactions by the knockout of phosphatases responsible for FPP hydrolysis and the optimisation of culture conditions [16,21]. As a result, we could observe decreased levels of *trans-trans-*farnesol in the GC-MS measurements of the corresponding phosphatase knockout strains (Figure 3), which was in accordance to the observation of Faulkner et al. [16] who reported on a higher in vitro activity of DPP1 compared to LPP1 and an additional effect regarding the double knockout of the phosphatases. However, a proportional increase of germacrene-A synthesis was only observed in the strain carrying the *LPP1* knockout, which is in accordance with the equivocal results of previous studies dealing with the knockout of these phosphatases in the context of enhanced isoprenoid synthesis in yeast [17,18,19,20]. As such, the double knockout of *LPP1* and *DPP1* was reported to result in the enhanced synthesis of α-santalene, farnesene and lycopene [19,20,34,35], whereas the reduced FPP hydrolysis upon *DPP1* knockout did not lead to the accumulation of corresponding sesquiterpenes in another study [17]. In this study, the authors therefore suggested that the potentially enhanced FPP pool in the *DPP1* knockout strain was responsible for feedback inhibition of the upper parts of the MVA-pathway, as an FPP-derived signal was described to be responsible for HMGR2 instability [36].

Additional results were obtained upon the use of different initial pH values during yeast cultivation. Thereby, an initial alkaline media (pH 8.5) decreased *trans-trans-*farnesol accumulation. In contrast, enhanced *trans-trans-*farnesol formation was observed upon the use of alkaline media in a study aiming at the enhanced formation of farnesol [21]. However, we exceeded the alkaline pH value used in the corresponding study. This possibly led to an overall decreased fitness and/or productivity of the used strain, due to an enhanced activity of the ATP-consuming plasma membrane H^+^-ATPase. An enhanced ATP consumption of the H^+^-ATPase that creates the pH and electrochemical gradient, driving the H^+^-dependent uptake of amino acids, sugars and inorganic ions, may compete with the ATP-dependent acetyl-CoA supply for the MVA-pathway in the alkaline culture media [37,38].

Nevertheless, we could further enhance germacrene-A biosynthesis by the use of acidic media for yeast cultivation. Therefore, the presented results that led to a strong germacrene-A biosynthesis underline the importance of metabolic engineering approaches together with the optimisation of culture conditions for enhanced isoprenoid synthesis in yeast.

## Supplementary Materials

The following are available online at https://www.mdpi.com/2306-5354/7/4/135/s1, Figure S1: Sequence alignments and sequencing data corresponding to the *LPP1* and *DPP1* knockout strains, Table S1: oligonucleotides used to generate the guide RNA expression cassette in pML104 plasmids or for stop codon integration, Table S2: Yeast strains used to enhance the metabolic flux towards FPP and the corresponding sterol- and farnesol/germacrene-A measurements.
