# Supplementary material for: Combinatorial Metabolic Engineering in Saccharomyces cerevisiae for the Enhanced Production of the FPP-Derived Sesquiterpene Germacrene
Source: Bioengineering (Basel). 2020 Oct 24;7(4):135. doi: 10.3390/bioengineering7040135 (PMC7712416; doi:10.3390/bioengineering7040135)
Supplement: Supplementary file 1 [file bioengineering-07-00135-s001.pdf]

bioengineering

MDPI

Supplementary Material of Combinatorial Metabolic Engineering in *Saccharomyces cerevisiae* for the Enhanced Production of the FPP-Derived Sesquiterpene Germacrene

a)

```
LPP1_WT         ATGATCTCTGTCATGGCGGATGAGAAACATAAGGAGTATTTTAAGCTATACTACTTTCAG
LPP1_delta      ATGATCTCTGTCATGGCGGATGAGAAACATAAGGAGTATTTTAAGCTATACTACTTTCAG
                ************************************************************

LPP1_WT         TACATGATAATTGGTCTATGTACGATATTATTCCTCTATTCGGAGATATCCCTGGTACCT
LPP1_delta      TACATGATAATTGGTCTATGTACGATATTATTCCTCTATTCGGAGATATCCCTGGTACCT
                ************************************************************

LPP1_WT         AGGGGCCAAAACATCGAATTTAGTCTTGATGACCCCAGTATATCAAAACGTTATGTACCT
LPP1_delta      AGGGGCCAAAACATCGAATTTAGTCTTGATGACCCCAGTATATCAAAACGTTATGTACCT
                ************************************************************

LPP1_WT         AACGAACTCGTGGGCCCACTAGAATGTTTGATTTTGAGTGTTGGACTGAGTAACATGGTC
LPP1_delta      AACGAACTCGTGGGCCCACTAGAATGTTTGATTTTGAGTGTTGGACTGAGTAACATGGTC
                ************************************************************

LPP1_WT         GTCTTCTGGACCTGCATGTTTGACAAGGACTTACTGAAGAAGAATAGAGTA-AAGAGACT
LPP1_delta      GTCTTCTGGACCTGCATGTTTGACAAGGACTTACTGAAGAAGAATAGAGTATAAGAGACT
                *************************************************** ********

LPP1_WT         AAGAGAGAGGCCGGACGGAATCTCGAACGATTTTCACTTCATGCATACTAGCATTCTATG
LPP1_delta      AAGAGAGAGGCCCGACGGAATCTCGAACGATTTTCACTTCATGCATACTAGCATTCTATG
                ************ ***********************************************

LPP1_WT         TCTGATGCTGATTATAAGCATAAATGCTGCCCTAACAGGCGCCTTAAAGTTGATTATAGG
LPP1_delta      TCTGATGCTGATTATAAGCATAAATGCTGCCCTAACAGGCGCCTTAAAGTTGATTATAGG
                ************************************************************

LPP1_WT         AAACTTGAGGCCTGACTTTGTTGATAGATGTATACCTGACCTCCAAAAGATGAGTGATTC
LPP1_delta      AAACTTGAGGCCTGACTTTGTTGATAGATGTATACCTGACCTCCAAAAGATGAGTGATTC
                ************************************************************

LPP1_WT         AGATTCTTTGGTTTTTGGCTTGGACATTTGCAAGCAGACTAACAAATGGATTCTATACGA
LPP1_delta      AGATTCTTTGGTTTTTGGCTTGGACATTTGCAAGCAGACTAACAAATGGATTCTATACGA
                ************************************************************
```

```
LPP1_WT         AGGCTTAAAAAGCACTCCAAGCGGACATTCAAGTTTCATAGTCAGTACCATGGGCTTTAC
LPP1_delta      AGGCTTAAAAAGCACTCCAAGCGGACATTCAAGTTTCATAGTCAGTACCATGGGCTTTAC
                ************************************************************

LPP1_WT         ATATCTTTGGCAAAGGGTTTTCACCACACGCAATACAAGAAGTTGCATTTGGTGCCCTTT
LPP1_delta      ATATCTTTGGCAAAGGGTTTTCACCACACGCAATACAAGAAGTTGCATTTGGTGCCCTTT
                ************************************************************

LPP1_WT         ATTAGCTCTAGTAGTAATGGTTTCAAGGGTTATCGATCACAGACATCATTGGTACGATGT
LPP1_delta      ATTAGCTCTAGTAGTAATGGTTTCAAGGGTTATCGATCACAGACATCATTGGTACGATGT
                ************************************************************

LPP1_WT         TGTCTCTGGAGCTGTTCTAGCATTTTTAGTCATTTATTGTTGCTGGAAATGGACATTTAC
LPP1_delta      TGTCTCTGGAGCTGTTCTAGCATTTTTAGTCATTTATTGTTGCTGGAAATGGACATTTAC
                ************************************************************

LPP1_WT         AAACTTGGCGAAAAGAGACATACTTCCTTCACCGGTTAGTGTTTAG
LPP1_delta      AAACTTGGCGAAAAGAGACATACTTCCTTCACCGGTTAGTGTTTAG
                **********************************************
```

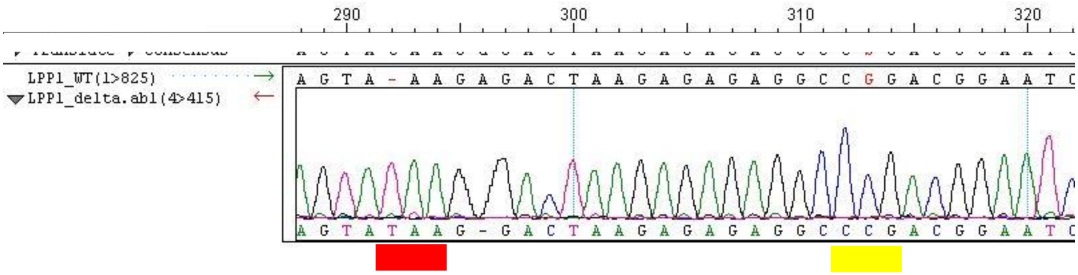

b)

```
DPP1_WT      ATGAACAGAGTTTCGTTTATTAAAACGCCTTTCAACATAGGGGCGAAATGGAGATTAGAA
DPP_delta    ATGAACAGAGTTTCGTTTATTAAAACGCCTTTCAACATAGGGGCGAAATGGAGATTAGAA
             ************************************************************

DPP1_WT      GATGTCTTTTTGCTCATTATCATGATACTTCTTAACTACCCAGTGTATTACCAACAACCG
DPP_delta    GATGTCTTTTTGCTCATTATCATGATACTTCTTAACTACCCAGTGTATTACCAACAACCG
             ************************************************************

DPP1_WT      TTCGAACGTCAGTTTTACATTAACGATCTCACTATATCGCATCCTTATGCGACAACTGAA
DPP_delta    TTCGAACGTCAGTTTTACATTAACGATCTCACTATATCGCATCCTTATGCGACAACTGAA
             ************************************************************

DPP1_WT      CGTGTAAATAACAACATGTTGTTTGTTTATAGTTTTGTCGTGCCATCTTTAACCATATTG
DPP_delta    CGTGTAAATAACAACATGTTGTTTGTTTATAGTTTTGTCGTGCCATCTTTAACCATATTG
             ************************************************************

DPP1_WT      ATAATTGGTTCCATTTTGGCCGATAGAAGACATTTGATT---TTTATTTTGTACACATCT
DPP_delta    ATAATTGGTTCCATTTTGGCCGATAGAAGACATTTGATTTAATTTATTTTGTACACATCT
             ***************************************   ******************

DPP1_WT      CTCCTTGGTTTATCACTCGCTTGGTTCAGTACGAGTTTCTTTACAAACTTCATCAAGAAT
DPP_delta    CTCCTTCGTTTATCACTCGCTTGGTTCAGTACGAGTTTCTTTACAAACTTCATCAAGAAT
             ****** *****************************************************

DPP1_WT      TGGATTGGAAGACTAAGACCAGATTTTCTAGATCGTTGCCAACCTGTTGAAGGCTTGCCA
DPP_delta    TGGATTGGAAGACTAAGACCAGATTTTCTAGATCGTTGCCAACCTGTTGAAGGCTTGCCA
             ************************************************************

DPP1_WT      TTGGACACTTTATTTACTGCAAAAGATGTGTGTACGACTAAGAATCACGAACGTCTGTTG
DPP_delta    TTGGACACTTTATTTACTGCAAAAGATGTGTGTACGACTAAGAATCACGAACGTCTGTTG
             ************************************************************

DPP1_WT      GATGGGTTTAGGACAACTCCGTCAGGTCATTCAAGTGAAAGCTTTGCAGGACTGGGTTAT
DPP_delta    GATGGGTTTAGGACAACTCCGTCAGGTCATTCAAGTGAAAGCTTTGCAGGACTGGGTTAT
             ************************************************************

DPP1_WT      TTGTACTTCTGGCTATGTGGGCAACTTTTGACTGAATCACCGTTGATGCCTTTATGGAGA
DPP_delta    TTGTACTTCTGGCTATGTGGGCAACTTTTGACTGAATCACCGTTGATGCCTTTATGGAGA
             ************************************************************
```

```
DPP1_WT         AAAATGGTGGCCTTTCTACCACTGTTAGGAGCTGCACTAATTGCTCTATCCAGAACTCAA
DPP_delta       AAAATGGTGGCCTTTCTACCACTGTTAGGAGCTGCACTAATTGCTCTATCCAGAACTCAA
                ************************************************************

DPP1_WT         GATTACAGACATCATTTCGTCGATGTAATTTTAGGGTCTATGTTGGGTTATATAATGGCA
DPP_delta       GATTACAGACATCATTTCGTCGATGTAATTTTAGGGTCTATGTTGGGTTATATAATGGCA
                ************************************************************

DPP1_WT         CACTTTTTCTACAGAAGAATCTTCCCACCCATTGATGATCCTCTTCCGTTCAAACCATTG
DPP_delta       CACTTTTTCTACAGAAGAATCTTCCCACCCATTGATGATCCTCTTCCGTTCAAACCATTG
                ************************************************************

DPP1_WT         ATGGACGATTCAGATGTCACCCTGGAGGAAGCAGTCACCCATCAGAGGATCCCGGATGAG
DPP_delta       ATGGACGATTCAGATGTCACCCTGGAGGAAGCAGTCACCCATCAGAGGATCCCGGATGAG
                ************************************************************

DPP1_WT         GAATTACATCCTTTGTCCGATGAAGGTATGTAA
DPP_delta       GAATTACATCCTTTGTCCGATGAAGGTATGTAA
                *********************************
```

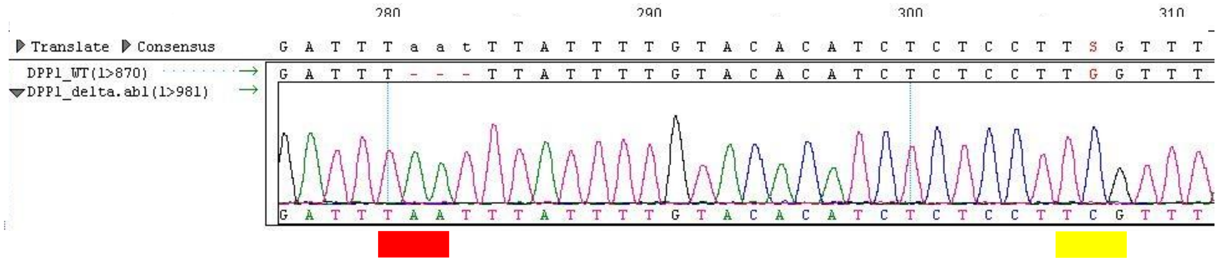

Figure S1. Sequence alignments and sequencing data corresponding to the *LPP1*- and *DPP1*-knockout strains. **a**) Sequence alignment and corresponding sequence data of the *S. cerevisiae LPP1* wildtype-allele (LPP1_WT) and *LPP1* knockout-allele (LPP1_delta) resulting from the integration of a premature stop codon. **b**) Sequence alignment and corresponding sequence data of the *S. cerevisiae DPP1* wildtype-allele (DPP1_WT) and *DPP1* knockout-allele (DPP1_delta) resulting from the integration of a premature stop codon. Red boxes indicate the integrated stop codon; yellow boxes indicate the modified PAM; underlined sequences correspond to the presented sequencing results; alignments of sequences were generated using the Clustal MUSCLE algorithm (http://www.ebi.ac.uk/Tools/msa/muscle/); Sequencing data were analysed using Lasergene SeqMan Pro (DNASTAR).

bioengineering

Table S1. Oligonucleotides used to generate the guide RNA expression cassette in pML104 plasmids or for stop codon integration. Italic letters indicate 20mer guide sequences; bold letters indicate the integrated stop codon; italic and bold letters indicate the modified protospacer-adjacent motif (PAM) to prevent further Cas9 activity.

Oligonucleotide Name	Sequence (5´to 3´)	Used for
***ROX1*-Guide RNA**	GAT C*TG AGG GGT CGA GTT AGC CCT* GTT TTA GAG CTA G	ligation in pML104
**P_{erg9}-Guide RNA**	GAT C*CG TGT TCT CGC TGC TCG TTT* GTT TTA GAG CTA G	ligation in pML104
***LPP1*-Guide RNA**	GAT C*AG ACT AAG AGA GAG GCC GGA* GTT TTA GAG CTA G	ligation in pML104
***DPP1*-Guide RNA**	GAT C*AT TTT GTA CAC ATC TCT CCT* GTT TTA GAG CTA G	ligation in pML104
***LPP1*-Stop**	TTT GAC AAG GAC TTA CTG AAG AAG AAT AGA GTA **TAA** GAG ACT AAG AGA GAG GC***C CG***A CGG AAT CTC GAA CGA TTT T	integration of stop codon
***DPP1*-Stop**	ATT TTG GCC GAT AGA AGA CAT TTG ATT **TAA** TTT ATT TTG TAC ACA TCT CTC CT***T CG***T TTA TCA CTC GCT TGG TTC AG	integration of stop codon

Table S2. Yeast strains used to enhance the metabolic flux towards sesquiterpenoid FPP and the corresponding sterol- and farnesol/germacrene-A measurements. Values are represented in g/g CDW.

Yeast Strain	Short Name Yeast Strain	Initial pH	$CuSO_4$ (μM)	Squalene	2,3-Oxidosqualene	lanosterol	Ergosterol	*Trans-Trans-*Farnesol	Germacrene-A
**WT**		6.5	0	0.0032 (±0.000877)	0.000015 (±0.000003)	0.00424 (±0.000691)	0.018667 (±0.001036)	-	-
**WT**<br>P_{GAL1}-*TkGAS2*	ST0	6.5	0	0.004219 (±0.00099)	0.000011 (±0.000002)	0.007663 (±0.001554)	0.020636 (±0.002723)	0.000716 (±0.000023)	0.000004 (±0.000006)
rox1::P_{GAL1}-*tHMGR* P_{GAL10}-*ERG13*	ST1	6.5	0	0.150902 (±0.044394)	0,000096 (±0.000031)	0.127558 (±0.043748)	0.082939 (±0.02365)	-	-
rox1::P_{GAL1}-*tHMGR* P_{GAL10}-*ERG13* P_{GAL1}-*TkGAS2*	ST3	6.5	0	0.077391 (±0.011625)	0.000063 (±0.000008)	0.029367 (±0.004729)	0.016594 (±0.002407)	0.003338 (±0.000284)	0.000393 (±0.000038)
rox1::P_{GAL1}-*tHMGR* P_{GAL10}-*ERG13*<br>$P_{erg9\Delta}$::P_{CTR3}	ST2	6.5	0	0.100485 (±0.011249)	0.000109 (±0.00003)	0.028349 (±0.003271)	0.038247 (±0.004749)	-	-
rox1::P_{GAL1}-*tHMGR* P_{GAL10}-*ERG13*<br>$P_{erg9\Delta}$::P_{CTR3}	ST2	6.5	150	0.001521 (±0.000204)	0.000782 (±0.000056)	0.003958 (±0.000602)	0.024172 (±0.002494)	-	-
rox1::P_{GAL1}-*tHMGR* P_{GAL10}-*ERG13*<br>$P_{erg9\Delta}$::P_{CTR3}	ST2	6.5	375	0.001337 (±0.00023)	0.001044 (±0.000239)	0.005181 (±0.001151)	0.022969 (±0.002797)	-	-
rox1::P_{GAL1}-*tHMGR* P_{GAL10}-*ERG13*<br>$P_{erg9\Delta}$::P_{CTR3}<br>P_{GAL1}-*TkGAS2*	ST4	6.5	150	0.001268 (±0.000067)	0.000387 (±0.000029)	0.004861 (±0.0001324)	0.011888 (±0.001612)	0.122461 (±0.003535)	0.001097 (±0.000044)
rox1::P_{GAL1}-*tHMGR* P_{GAL10}-*ERG13*<br>$P_{erg9\Delta}$::P_{CTR3}<br>*lpp1Δ*<br>P_{GAL1}-*TkGAS2*	ST5	4.5	15	0.002755 (±0.000917)	0.003491 (±0.000512)	0.007104 (±0.001601)	0.03149 (±0.005712)	0.11631 (±0.003323)	0.007454 (±0.000317)
rox1::P_{GAL1}-*tHMGR* P_{GAL10}-*ERG13*<br>$P_{erg9\Delta}$::P_{CTR3}<br>*lpp1Δ*<br>P_{GAL1}-*TkGAS2*	ST5	6.5	150	0.001315 (±0.000284)	0.001117 (±0.000549)	0.003273 (±0.000744)	0.013671 (±0.001356)	0.111314 (±0.003375)	0.00414 (±0.00015)
rox1::P_{GAL1}-*tHMGR* P_{GAL10}-*ERG13*<br>$P_{erg9\Delta}$::P_{CTR3}<br>*lpp1Δ*<br>P_{GAL1}-*TkGAS2*	ST5	8.5	150	0.041856 (±0.005932)	0.000837 (±0.000153)	0.02078 (±0.003467)	0.038452 (±0.004871)	0.032799 (±0.009024)	0.003457 (±0.001213)
rox1::P_{GAL1}-*tHMGR* P_{GAL10}-*ERG13*<br>$P_{erg9\Delta}$::P_{CTR3}<br>*dpp1Δ*<br>P_{GAL1}-*TkGAS2*	ST6	6.5	150	0.00102 (±0.000091)	0.000732 (±0.000117)	0.003713 (±0.002224)	0.009354 (±0.003776)	0.091141 (±0.002983)	0.000922 (±0.000069)
rox1::P_{GAL1}-*tHMGR* P_{GAL10}-*ERG13*<br>$Pe_{rg9\Delta}$::P_{CTR3}<br>*lpp1Δ dpp1Δ*<br>P_{GAL1}-*TkGAS2*	ST7	6.5	150	0.000961 (±0.00032)	0.000545 (±0.003278)	0.002599 (±0.001472)	0.013053 (±0.003278)	0.048922 (±0.005419)	0.000743 (±0.000307)

bioengineering

Publisher's Note: MDPI stays neutral with regard to jurisdictional claims in published maps and institutional affiliations.

© 2020 by the authors. Submitted for possible open access publication under the terms and conditions of the Creative Commons Attribution (CC BY) license (http://creativecommons.org/licenses/by/4.0/).
